# Successful radioimmunotherapy of established syngeneic rat colon carcinoma with ^211^At-mAb

**DOI:** 10.1186/2191-219X-3-23

**Published:** 2013-04-04

**Authors:** Sophie E Eriksson, Tom Bäck, Erika Elgström, Holger Jensen, Rune Nilsson, Sture Lindegren, Jan Tennvall

**Affiliations:** 1 Division of Oncology, Department of Clinical Sciences, Lund University, Barngatan 2B, Lund 221 85, Sweden; 2 Department of Radiation Physics, Institute of Clinical Sciences, Sahlgrenska Academy, University of Gothenburg, Gothenburg, 413 45, Sweden; 3 Cyclotron and PET Unit, Rigshospitalet, Copenhagen, 2100, Denmark; 4 Department of Oncology, Skåne University Hospital, Lund, 221 85, Sweden

**Keywords:** Radioimmunotherapy, Astatine-211, Targeted alpha therapy, Colon carcinoma

## Abstract

**Background:**

Most carcinomas are prone to metastasize despite successful treatment of the primary tumor. One way to address this clinical challenge may be targeted therapy with α-emitting radionuclides such as astatine-211 (^211^At). Radioimmunotherapy utilizing α-particle emitting radionuclides is considered especially suitable for the treatment of small cell clusters and single cells, although lesions of different sizes may also be present in the patient. The aim of this study was primarily to evaluate the toxicity and secondarily *in vivo* efficacy of a ^211^At-labeled monoclonal antibody (mAb) directed against colon carcinoma with tumor diameters of approximately 10 mm.

**Methods:**

Eighteen rats with subperitoneal syngeneic colon carcinoma were allocated to three groups of six animals together with three healthy rats in each group. The groups were injected intravenously with either 150 μg of unlabeled mAbs (controls) or 2.5 or 5 MBq ^211^At-mAbs directed towards the Lewis Y antigen expressed on the cell membrane of several carcinomas. Tumor volume, body weight, and blood cell counts were monitored for 100 days after treatment.

**Results:**

Local tumors were non-palpable in five out of six rats after treatment with both activities of ^211^At-mAbs, compared to one out of six in the control group. At the study end, half of the animals in each group given ^211^At-BR96 and one animal in the control group were free from disease. Radioimmunotherapy resulted in dose-dependent, transient weight loss and myelotoxicity. Survival was significantly better in the groups receiving targeted alpha therapy than in those receiving unlabeled mAbs.

**Conclusions:**

This study demonstrates the possibility of treating small, solid colon carcinoma tumors with α-emitting radionuclides such as ^211^At bound to mAbs, with tolerable toxicity.

## Background

Although the treatment of primary tumors is often successful, metastatic disease is the major cause of cancer-related mortality [1]. There is thus a need for new treatment modalities targeting metastases in order to improve the survival of patients suffering from malignant tumors.

During radioimmunotherapy, radionuclides are directed to tumor lesions by specific targeting using monoclonal antibodies (mAbs) as carrier molecules. The concept has resulted in two FDA-approved radioimmunoconjugates, ^90^Y-ibritumomab tiuxetan and ^131^I-tositumomab, for the treatment of non-Hodgkin's lymphoma. However, several studies have shown radioimmunotherapy to be less effective in the treatment of larger solid tumors [2,3], and the focus has therefore changed to treating small lesions including metastases [4,5]. Due to the relatively long path length of β-particles, β-emitting radionuclides are generally considered unsuitable for targeting microscopic tumors as much of the radiation will be deposited outside the tumor. Alpha-emitting radionuclides have a much shorter particle path length (typically <100 μm) resulting in less irradiation of healthy tissue [6]. One such α-emitting radionuclide is ^211^At, which has a half-life of 7.2 h and a particle range in soft tissue of 55 to 80 μm. Promising results have been obtained in preclinical studies on ^211^At-labeled mAbs in models of, e.g., leukemia [7,8] and ovarian [9-11] and colon carcinoma [12], and in clinical studies of glioma and ovarian carcinoma [13,14].

Colon carcinoma is among the most common cancer diseases and is prone to metastasize [15]. *In vivo* studies on radioimmunotherapy with the β-emitting radionuclide ^177^Lu (maximal range in soft tissue, 1.8 mm) with the aim of treating colon carcinoma metastases resulted in prolonged survival [16,17]. The primary aim of the present study was to evaluate the toxicity and secondly the therapeutic efficacy of ^211^At-labeled mAb on small solid tumors of colon carcinoma in a syngeneic immunocompetent rat model. To the best of our knowledge, ^211^At-mAbs have not previously been evaluated in an immunocompetent animal model. The same animal model and mAb have previously been utilized for studies of radioimmunotherapy with the β-emitting radionuclides ^177^Lu and ^90^Y [18,19].

## Methods

### Monoclonal antibody

BR96 (Seattle Genetics Inc., Seattle, WA, USA) is a chimeric (mouse/human) IgG1 mAb recognizing the Lewis Y epitope. Lewis Y is expressed in several carcinomas, including breast, gastrointestinal, pancreatic, non small cell lung, cervical, and ovarian cancer, and in some melanomas. As with many tumor-associated antigens, the epitope is also expressed in some normal tissues, including the epithelial cells of the gastrointestinal tract in humans [20] and in the rat strain used in this study [19].

### Radiochemistry

Astatine-211 was produced by irradiating stable bismuth using the ^209^Bi(α,2n)^211^At reaction at the Cyclotron and PET Unit, Rigshospitalet, Copenhagen, Denmark. After irradiation, the target was transported to the Department of Nuclear Medicine at Sahlgrenska University Hospital, Gothenburg, Sweden, where the astatine was transformed into a chemically useful form by dry distillation, as described previously [21].

^211^At labeling of BR96 was performed essentially as described previously [22]. Briefly, the antibody was first reacted with the *N*-succinimidyl-3-(trimethylstannyl) benzoate reagent to give the ε-lysyl-3-(trimethylstannyl)benzamide BR96 immunoconjugate. After 30 min of incubation with the reagent, the BR96 conjugate was isolated in 0.1 M citrate buffer (pH 5.5) using a Sephadex NAP-5 column. From the BR96 conjugate preparation, 800 μg was added to a vial containing 336 MBq of ^211^At oxidized by 15 μL *N*-iodosuccinimide (NIS; 67 μM) in methanol with 1% acetic acid during vigorous agitation. After 1 min, 3 μL NIS (1 mg/mL) was added, and the reaction mixture was incubated for 1 min before terminating the reaction with 5 μL sodium ascorbate (50 mg/mL). The antibody fraction, ^211^At-BR96, was isolated using a Sephadex NAP-5 column. To protect the antibody from radiolysis, the 0.9-mL product volume was eluted into a vial containing 0.1 mL phosphate buffered saline (PBS) with 10% bovine serum albumin. Unlabeled BR96 was added to adjust the mAb dose to 150 μg per animal.

The antigen-binding properties (immunoreactivity) of ^211^At-BR96 relative to BR96 were analyzed by determination of the equilibrium binding constant (*K*_d_) by saturation binding curve analysis as previously described [18], using BN7005 cells as the target antigen. The immunoreactivity is given by the ratio *K*_d_(BR96)/*K*_d_(^211^At-BR96).

### Animal model

The BN7005-H_1_D_2_ cell line was originally established from a colon carcinoma detected in a 1,2-dimethylhydrazine-treated Brown Norway rat. The cell line expresses the Lewis Y antigen both *in vitro* and *in vivo*. The survival fraction after external irradiation with 2 Gy has been determined to be 0.5, which indicates moderate radiosensitivity comparable to human colon carcinoma cell lines [23]. Cells were cultured in RPMI 1640 medium supplemented with 10% fetal bovine serum, 1 mM sodium pyruvate, 10 mM Hepes buffer (all from PAA Laboratories GmbH, Pasching, Austria), and 14 mg/L gentamicin (Gibco, Invitrogen, Carlsbad, CA, USA) at 37°C in a humidified environment before harvesting by trypsin treatment and suspension in supplemented medium prior to inoculation.

Immunocompetent male Brown Norway rats (Harlan Laboratories, Horst, the Netherlands), with a body weight of approximately 250 g, were inoculated with 3 × 10^5^ BN7005-H_1_D_2_ cells in 50 μL culturing medium between the peritoneum and the abdominal muscle under anesthesia with isoflurane (Abbott Scandinavia AB, Solna, Sweden). Tumor growth was monitored by palpation and tumor measurement with a digital caliper. The animals were housed under standard conditions and fed pellets and fresh water *ad libitum*. The experiment was approved by the regional animal ethics committee and followed Swedish legislation on animal welfare and protection.

### Dosimetry and estimation of maximum tolerable activity

The activities of ^211^At in this study were chosen with respect to the absorbed dose to the bone marrow. In a review on the toxicity of α-emitting radionuclides, Dahle et al. [24] presented values for maximal tolerable doses to the bone marrow (MTD_BM_) ranging from 0.4 to 7.6 Gy for different α-emitters. Intravenous (i.v.) administration of ^211^At-labeled antibodies to mice indicated values of MTD_BM_ from 2 to 4 Gy [25,26]. A value of 4 Gy was chosen as the MTD_BM_ in the present study, and the absorbed dose to the bone marrow was estimated using bone marrow uptake data from a previous biodistribution study of ^177^Lu-BR96 in the same animal model [27]. Using these ^177^Lu-BR96 uptake data (% injected activity/g tissue), theoretical time-activity curves were obtained for ^211^At-BR96 and used to calculate the cumulated activity (i.e., the total number of decays, *Ã*) of ^211^At. Assuming an absorbed fraction of the α-particles, *ϕ*_*α*_, to be 1 and including contributions only from α-particles, the absorbed dose (*D*) was calculated using the following equation:

$$D=\frac{Ã}{m}\Delta_{\alpha }\phi_{\alpha }$$

where *m* denotes tissue mass, and Δ_α_ is the mean energy released per ^211^At decay (here assumed to be 1.09 × 10^−12^ J). The estimated mean absorbed dose to the bone marrow for ^211^At-BR96 was found to be 0.8 Gy/MBq. Using the assumed MTD_BM_ of 4 Gy, the corresponding maximum tolerable activity that could be injected was 5 MBq.

### Radioimmunotherapy

Two weeks (defined here as day 0) after inoculation, the tumor-bearing rats were allocated to three groups (six rats per group) with similar distributions of tumor size, see Table 1. In addition, three rats without tumors were included in each group to monitor toxicity without influence from tumor burden. The rats were injected with either 150 μg of unlabeled BR96 in PBS (control group), 2.5 MBq ^211^At-BR96 corresponding to 9 MBq/kg body weight (2.5 MBq group), or 5 MBq ^211^At-BR96 corresponding to 19 MBq/kg body weight (5 MBq group). The mAbs were administered via the tail vein under anesthesia, and the injection volume was 0.4 mL.

**Table 1:** Group characteristics at day 0 and injected activities (average values and ranges)

**Treatment group**	**Body weight (g)**	**Tumor volume (mm**^**3**^**)**	**Injected activity (MBq)**	**IA/kg body weight (MBq/kg)**
Controls (unlabeled BR96)				
Tumor (*n* = 6)	290 (282 to 300)	520 (282 to 640)	-	-
Non-tumor (*n* = 3)	289 (281 to 296)	-	-	-
2.5 MBq ^211^At-BR96				
Tumor (*n* = 6)	272 (269 to 278)	475 (256 to 640)	2.5 (2.5 to 2.6)	9.3 (9.0 to 9.6)
Non-tumor (*n* = 3)	276 (271 to 278)	-	2.4 (2.4 to 2.4)	8.8 (8.7 to 8.9)
5 MBq ^211^At-BR96				
Tumor (*n* = 6)	259 (247 to 266)	371 (176 to 520)	5.0 (4.9 to 5.1)	19.2 (18.3 to 19.9)
Non-tumor (*n* = 3)	264 (259 to 267)	-	4.8 (4.6 to 4.9)	18.0 (17.5 to 18.3)

IA, injected activity.

### Monitoring after treatment

Tumor sizes were measured twice weekly after treatment. Tumor volumes were calculated as tumor length × width^2^ × 0.4. Tumors not palpable for at least one consecutive week were classified as undetectable. Body weight was also recorded twice per week. Bone marrow toxicity was monitored by counting red blood cells, white blood cells, and platelets in arterial blood samples with a Vet CA530 Medonic Cell Analyzer (Boule Medical, Stockholm, Sweden) twice a week for the first 4 weeks, then once weekly until the end of the study. Plasma was sampled from animals without tumors for the analysis of liver and kidney function markers.

Animals were monitored up to 100 days after treatment as this was sufficient for detection of metastases in previous work with animals followed up to 180 days post injection (p.i.) [19]. The rats were sacrificed with an overdose of isoflurane at the end of the study or when the tumor exceeded 20 × 20 mm, body weight decreased by >15%, or if the animal's general health was affected. At the time of sacrifice, all animals were dissected systematically by the same person, and the number and location of metastatic sites were noted. Tumor findings detected at autopsy were fixed in 4% paraformaldehyde and embedded in paraffin.

### Immunohistochemistry

The tumor findings were sectioned and stained with hematoxylin and eosin for histological evaluation or examined immunohistochemically to evaluate BR96 target antigen expression and proliferation. In short, sections were dehydrated, and antigen retrieval was performed by heating the slides in citrate buffer, pH 6. After washing in Tris-buffered saline with 0.25% Tween 20 and blocking of endogenous peroxidases with Peroxidase Blocking Solution (Dako, Glostrup, Denmark), the sections were incubated with 5 μg/mL BR96 in Antibody Diluent (Dako) overnight or with Rabbit anti Ki67 (Clone SP6, NeoMarkers, Fremont, CA, USA) for 2 h, all at room temperature in a moist chamber. After washing, the secondary antibody donkey F(ab)_2_ anti-human IgG-HRP (BR96) or donkey anti-rabbit HRP (Ki67; both from Jackson ImmunoResearch Laboratories, West Grove, PA, USA) in Antibody Diluent was added to the sections and incubated for 3 h at room temperature. Finally, diaminobenzidine (Dako) was added before dehydration and mounting. The target antigen expression was evaluated in relation to tumor histology by estimating the approximate percentage of viable tumor cells with complete cell membrane staining.

### Statistical analyses

Prism 5.04 (GraphPad Software Inc., La Jolla, CA, USA) was used for statistical analyses. Weight loss and blood cell counts were analyzed with either one-way analysis of variance (ANOVA) or two-way ANOVA with Bonferroni multiple comparison post tests. Survival was analyzed with the log-rank Mantel-Cox test.

## Results

### Radiochemistry

The radiochemical purity (RCP) was 97% according to methanol precipitation. The non-decay-corrected radiochemical yield (RCY) was determined to be 78%, using the relation

$$\text{RCY}=\frac{A_{\text{tot}}\;\times \;\text{RCP}}{A_{\text{added}}}$$

where *A*_tot_ is the total activity eluted from the NAP-5 column, and *A*_added_ is the activity added to the reaction. The immunoreactivity, expressed as the ratio of the *K*_d_ for BR96 and ^211^At-BR96, was 0.93 for both specific activities. The *K*_d_ of ^211^At-BR96 was within the 95% confidence interval of the *K*_d_ for BR96.

### Toxicity

**Figure 1:**
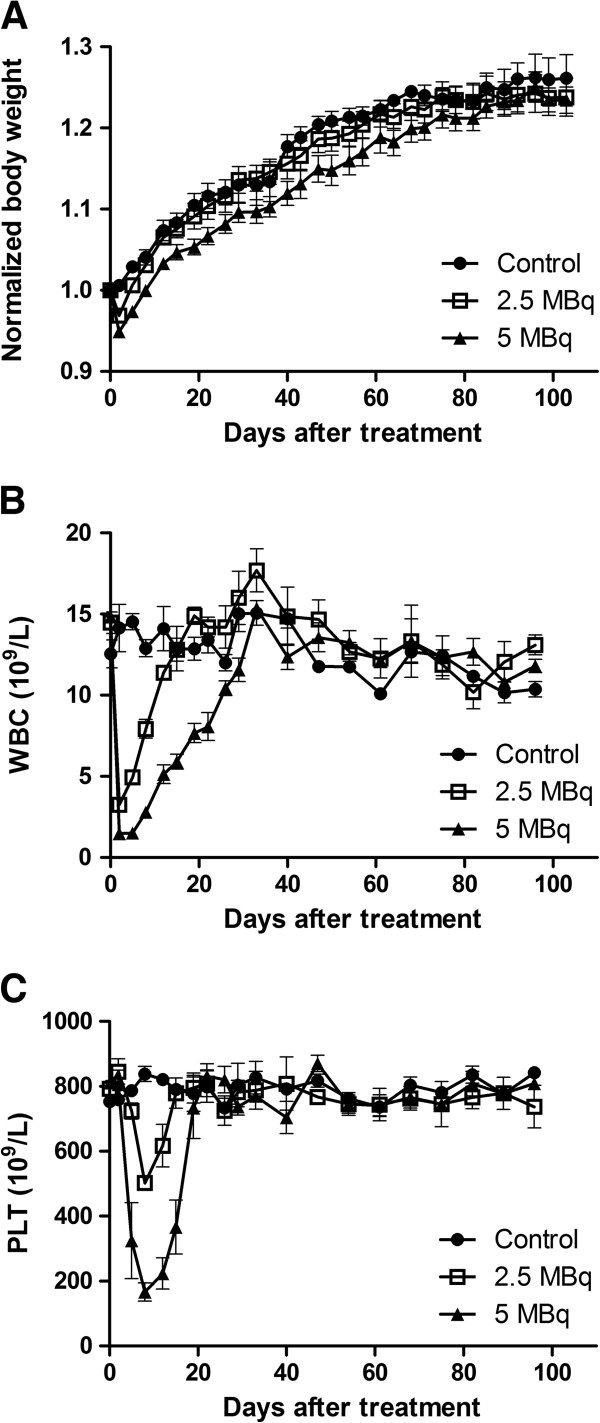
**Toxicity after i.v. administration of**^**211**^**At-BR96.** Body weight normalized to day 0 (**A**), white blood cell count (**B**), and platelet count (**C**). Error bars denote the standard error of the mean after intravenous injection of ^211^At-BR96. Animals with inoculated tumors were excluded from (**B**) and (**C**) in order to evade effects from tumor burden being mistaken as toxicity.

The administration of 2.5 and 5 MBq ^211^At-BR96 resulted in dose-dependent relative weight losses of 3% (range 1% to 6%) and 5% (range 4% to 8%), respectively, the nadir being seen on the first recording after treatment (day 2 p.i.). This can be compared to a weight increase of 1% in the control group (range −1% to 1%; *p* < 0.0001). The animals in the 2.5 MBq group showed full recovery of weight within 1 week, whereas the animals in the 5 MBq group showed a delayed progression in weight compared to the control group (difference not statistically significant) (Figure 1A).

The white blood cell counts decreased in a dose-dependent manner; the nadir being seen on day 2 (first sampling occasion) (*p* < 0.0001). Full recovery of white blood cell counts was observed on days 19 (median, range 15 to 22) and 33 (26 to 33) in the 2.5 and 5 MBq groups, respectively (Figure 1B). On day 8, the platelet count had decreased to approximately 50% of the baseline in the 2.5 MBq group and to 25% in the 5 MBq group (*p* < 0.0001) (Figure 1C). Platelet counts had recovered on day 19 (range 15 to 22) in the 2.5 MBq group and on day 22 (range 19 to 26) in the 5 MBq group. In one rat in each group given ^211^At, the number of platelets started to decrease after initial recovery and then remained at 25% to 50% of the initial value until the end of the study. There was no statistically significant variation in red blood cell counts between the three groups (data not shown). Data from rats with and without inoculated tumors were included in the statistical analyses.

The levels of alanine transaminase (ALAT) and gamma glutamyl transpeptidase (γGT) in plasma were used as markers of liver function. ALAT remained unaffected by treatment, whereas the level of γGT was elevated less than 2.5 times the upper limit of normal level (grade 1 according to the National Cancer Institute Common Terminology for Adverse Events version 4.0) for 3 weeks after the administration of the higher activity of ^211^At-BR96. The plasma levels of creatinine did not reveal any kidney toxicity after radioimmunotherapy with ^211^At-BR96.

### Treatment outcome

**Figure 2:**
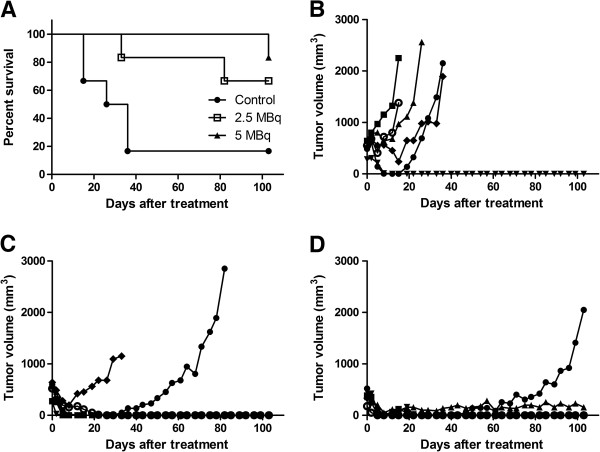
**Survival and tumor growth after treatment. **Survival of rats with established colon carcinoma tumors after treatment with ^211^At-BR96 (**A**). Individual tumor volumes after treatment with unlabeled BR96 (**B**), 2.5 MBq ^211^At-BR96 (**C**), and 5 MBq ^211^At-BR96 (**D**).

Survival was significantly prolonged in groups given ^211^At-BR96 compared to the control group (*p* = 0.017) (Figure 2A). Undetectable tumors were recorded in five out of six animals in both groups given ^211^At-BR96 and in one out of six animals in the control group, see Table 2. Remaining tumors continued to grow except in one animal in the 5 MBq group, which remained stable until the end of the study (Figure 2B,C,D). One recurrent local tumor was detected in each group given ^211^At-BR96, in both cases after 3 weeks of non-palpable tumors. In all groups, smaller tumors seemed to respond better to the therapy, as has been observed by others [28,29].

**Table 2:** **Tumor response after treatment with **^**211**^**At-BR96**

**Treatment group**	**Undetectable tumors**	**Stable tumors**	**Progressing tumors**	**Recurrence**	**Metastases at autopsy**
Controls (unlabeled BR96)	1/6	0/6	5/6	0/1	5/6
2.5 MBq ^211^At-BR96	5/6	0/6	1/6	1/5	3/6
5 MBq ^211^At-BR96	5/6	1/6	0/6	1/5	2/6

Metastases were found in all treatment groups, most commonly in the lymph nodes, but also in the liver and spread throughout the abdomen. Metastases were detected at autopsy in five animals in the control group that were sacrificed between days 16 and 35 due to high primary tumor burden. In the 2.5 MBq group, one animal with a remaining primary tumor sacrificed on day 33 due to signs of metastatic disease and one that was sacrificed on day 82 due to the burden of the recurrent tumor showed metastases at autopsy. Another rat in this group, sacrificed at the end of the study without any primary or recurrent tumor, was found to have a lymph node metastasis. In the 5 MBq group, one rat with recurrent disease sacrificed due to tumor burden at the end of the study was found to have two lymph node metastases, while one rat with stable disease was free from detectable metastases. An additional rat in this group, without a primary tumor, was found to have a lymph node metastasis at the end of the study.

**Figure 3:**
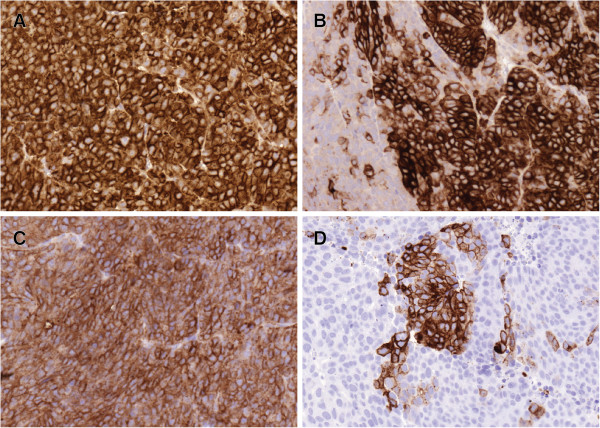
**Antigen expression after treatment. **The target antigen detected in a tumor treated with unlabeled BR96 (**A**), a tumor treated with 2.5 MBq ^211^At-BR96 (**B**), an abdominal metastasis in a rat treated with 2.5 MBq ^211^At-BR96 (**C**), and a lymph node metastasis in a rat treated with 5 MBq ^211^At-BR96 (**D**). (**B**) and (**D**) are from the same rat.

All tumor findings contained viable and proliferating tumor cells. Both primary tumors and detected metastases in the control group showed high expression of the BR96 target antigen, with complete membrane staining of at least >50% of all tumor cells; in half of the samples, the number of stained cells was >90% (Figure 3A). The target antigen was detected in more than 50% of the cells in half of the specimens from the two astatine-treated groups (Figure 3B,C,D). No tumor finding lacked expression of the target antigen; however, the number of positive cells was less than 10% in one-third of the tissues. The antigen was commonly detected in larger areas rather than on single cells surrounded by negative cells. Areas with weak staining were observed in several of the tissue sections. There were no trends regarding number of antigen-expressing tumor cells regarding the metastatic site. The two recurred primary tumors had lower expression compared to the stable and progressing tumors.

## Discussion

In this animal study, the concept of targeted alpha therapy was shown to be both tolerable and efficient in the treatment of established colon carcinoma. The activities administered were based on an estimate of the maximal tolerable dose to bone marrow, using historical ^177^Lu-BR96 biodistribution data for the theoretical calculation of the absorbed dose resulting from treatment with ^211^At-BR96 [27]. Both administered activities, 2.5 and 5 MBq/animal (9 and 19 MBq/kg body weight), were tolerated with regard to myelotoxicity, kidney, liver, and general toxicity. The minor tendency towards increased hematologic toxicity and the delayed weight progression in the group given 5 MBq indicate that this activity was probably close to the maximal tolerable activity.

The treatment resulted in undetectable tumors in five out of six rats in both groups treated with ^211^At-BR96. Survival was prolonged compared to the control group given unlabeled mAb. The increased survival in the group given 5 MBq ^211^At-BR96 was not significantly different from that in the group given 2.5 MBq. Due to the efficacy of the administration of 2.5 MBq, it is difficult to motivate the use of higher activity.

Assuming a heterogeneous tumor uptake of the antibody, as has recently been found for ^177^Lu-BR96 [30], due to physiological factors such as the distribution of functional blood vessels and high interstitial fluid pressure [31], eradication of all viable tumor cells by short-range α-particles could be impaired. The limited range may be compensated to some extent by the bystander effect [32]. Irradiation of the blood vessels resulting in compromised nutrition may also enhance the antitumor effect [33-35].

Targeted alpha therapy is generally regarded as being more appropriate for loco-regional treatment since the short physical half-life and the kinetics after i.v. administration of intact mAbs could mean that most decays have occurred before binding to the target antigen on tumor cells [36]. This also applies to our animal model, in which the maximal tumor uptake was observed 24 h p.i. [30]. Therefore, the strong antitumor response, resulting in undetectable tumors in five out of six animals in both groups given ^211^At-mAb seen in the present study, was not expected. Recent mouse studies on i.v. therapy of small solid tumors using ^211^At-labeled antibodies [37] indicated that mean absorbed tumor doses above 10 Gy were required for the eradication of small solid tumors. No experimental data on tumor uptake of ^211^At-BR96 were available for the estimation of the absorbed dose to the tumors in the present study. However, using tumor uptake data for ^177^Lu-BR96, the estimated mean absorbed dose to the tumors would be approximately 9.6 and 4.8 Gy, for activities of 5 and 2.5 MBq, respectively.

The antitumor response could have been enhanced by the mAb itself due to the initiation of antibody-dependent cell-mediated cytotoxicity and/or complement-dependent cytotoxicity [38,39]. The presence of such effects was demonstrated in the control group, in which one tumor disappeared completely and four other tumors showed a small initial response, as has been seen previously in our animal model [30]. An advantage of syngeneic immunocompetent animal models is the possibility to study the synergistic effects of radioimmunotherapy and the adaptive immune system.

Metastases were detected in five out of six rats treated with unlabeled BR96, despite the shorter survival time, and in approximately half of the animals in the groups given ^211^At-BR96. All metastases demonstrated antigen expression, but to a much lower extent in the groups given ^211^At-BR96. This may be the result of the elimination of tumor cells with sustained antigen expression and repopulation by clones of cells lacking the target antigen, thereby reducing the possibility of eradicating metastases by repeating the treatment. It is currently not known whether tumor cells are disseminated and spread before or during radioimmunotherapy in our animal model since the untreated animals are often sacrificed before any metastases can be detected due to heavy primary tumor burden.

The targeted alpha therapy using ^211^At-labeled mAbs in this study resulted in a comparable rate of CR in tumor-bearing rats to that found when using ^177^Lu-labeled BR96 in the same rat tumor model [18]. The proportion of animals with detectable disseminated disease was around 50% after both α and β radioimmunotherapy. Beta-emitting radionuclides are generally regarded as being better suited for the treatment of solid tumors due to their longer particle range than α-emitting radionuclides, reducing the effect of heterogeneous tumor distribution. However, this property limits the effectiveness against small cell clusters and singe cells [40] as most of the energy is deposited outside the tumor. One therapeutic strategy could be to combine α- and β-emitting radionuclides to utilize their different properties. It would also be relevant to develop an orthotopic metastasis model with our syngeneic system, with smaller tumor lesions in the liver and/or lungs instead of the comparably larger solid tumors used in the present study. Such a study on microscopic metastases in the liver has been performed with ^177^Lu-labeled mAbs in a model of colon cancer [16]. The results of that study showed increased survival and delayed tumor growth in animals treated with radioimmunotherapy, but the cure rate was not affected. A comparison of α and β radioimmunotherapy in such a model would be useful in providing evidence of the suitability of the different radionuclides regarding their physical properties in the treatment of metastases.

## Conclusions

Treatment with ^211^At-mAbs was tolerable with respect to toxicity at activity levels resulting in undetectable tumors in a syngeneic rat colon carcinoma model. The results demonstrate that radioimmunotherapy with ^211^At can be an effective treatment modality for well-established and well-vascularized tumors up to a size of 10 to 15 mm, but did not affect the development of metastatic disease.

## Competing interests

The authors declare that they have no competing interests.

## Authors’ contributions

SEE designed and performed the *in vivo* studies, evaluated the immunohistochemistry, performed the statistical analyses, and wrote the manuscript. TB participated in planning the experiment, performed the dosimetric calculations, and contributed to data interpretation and manuscript writing. EE participated in planning and performing the *in vivo* studies. HJ developed and performed production of ^211^At. SL developed and performed the radiolabeling, and contributed to data interpretation and manuscript writing. JT and RN contributed to the study design and analyses of data as well as writing of the manuscript. All authors read and approved the final manuscript.
